# Stable isotopes can be used to infer the overwintering locations of prebreeding marine birds in the Canadian Arctic

**DOI:** 10.1002/ece3.3410

**Published:** 2017-09-18

**Authors:** Rolanda J. Steenweg, Glenn T. Crossin, T. Kurt Kyser, Flemming R. Merkel, H. Grant Gilchrist, Holly L. Hennin, Gregory J. Robertson, Jennifer F. Provencher, Joanna Mills Flemming, Oliver P. Love

**Affiliations:** ^1^ Department of Biology Dalhousie University Halifax NS Canada; ^2^ Department of Geological Sciences and Geological Engineering Queen's University Kingston ON Canada; ^3^ Greenland Institute of Natural Resources Nuuk Greenland; ^4^ Department of Bioscience Aarhus University Roskilde Denmark; ^5^ Environment and Climate Change Canada National Wildlife Research Centre Carleton University Ottawa ON Canada; ^6^ Department of Biological Sciences Great Lakes Institute for Environmental Research University of Windsor Windsor ON Canada; ^7^ Environment and Climate Change Canada Wildlife Research Division Mount Pearl NL Canada; ^8^ Department of Biology Acadia University Wolfville NS Canada; ^9^ Department of Mathematics and Statistics Dalhousie University Halifax NS Canada

**Keywords:** biomarker, carbon, carryover, claw, *k*‐means cluster analysis, nitrogen, population delineation, seabird, toenail, tracking

## Abstract

Although assessments of winter carryover effects on fitness‐related breeding parameters are vital for determining the links between environmental variation and fitness, direct methods of determining overwintering distributions (e.g., electronic tracking) can be expensive, limiting the number of individuals studied. Alternatively, stable isotope analysis in specific tissues can be used as an indirect means of determining individual overwintering areas of residency. Although increasingly used to infer the overwintering distributions of terrestrial birds, stable isotopes have been used less often to infer overwintering areas of marine birds. Using Arctic‐breeding common eiders, we test the effectiveness of an integrated stable isotope approach (13‐carbon, 15‐nitrogen, and 2‐hydrogen) to infer overwintering locations. Knowing the overwinter destinations of eiders from tracking studies at our study colony at East Bay Island, Nunavut, we sampled claw and blood tissues at two known overwintering locations, Nuuk, Greenland, and Newfoundland, Canada. These two locations yielded distinct tissue‐specific isotopic profiles. We then compared the isotope profiles of tissues collected from eiders upon their arrival at our breeding colony, and used a *k*‐means cluster analysis approach to match arriving eiders to an overwintering group. Samples from the claws of eiders were most effective for determining overwinter origin, due to this tissue's slow growth rate relative to the 40‐day turnover rate of blood. Despite taking an integrative approach using multiple isotopes, *k*‐means cluster analysis was most effective when using 13‐carbon alone to assign eiders to an overwintering group. Our research demonstrates that it is possible to use stable isotope analysis to assign an overwintering location to a marine bird. There are few examples of the effective use of this technique on a marine bird at this scale; we provide a framework for applying this technique to detect changes in the migration phenology of birds' responses to rapid changes in the Arctic.

## 1 INTRODUCTION

The nonbreeding phase of the annual cycle is increasingly being recognized for its impacts on individual fitness in animals, as many physiological, behavioral, and life‐history‐related traits that influence breeding phenology and investment are shaped by the selective pressures operating at this time, which can generate carryover effects (Greenberg & Marra, 2005; Williams, 2012). The study of carryover effects—especially how variation in overwintering experiences can impact subsequent reproductive performance, population processes, and fitness—is a burgeoning field of research that is central to testing hypotheses of behavioral, evolutionary, and physiological ecology (O'Connor, Norris, Crossin, & Cooke, 2014)**.** Individual variation in overwintering location impacts foraging activity and physiological condition at arrival on the breeding grounds, and therefore an individual's preparedness for breeding (Descamps, Bêty, Love, & Gilchrist, 2011; Marra, Hobson, & Holmes, 1998; Sorensen, Hipfner, Kyser, & Norris, 2009). As arrival condition and timing on the breeding grounds are key traits known to impact the breeding phenology and success of migratory birds (Bêty, Gauthier, & Giroux, 2003; Gunnarsson, Gill, Newton, Potts, & Sutherland, 2005; Hennin et al., 2016; Love, Gilchrist, Descamps, Semeniuk, & Bêty, 2010), determining how an individual's winter experience affects these traits is a key step in understanding population‐level processes.

Analysis of naturally occurring biochemical markers in tissues is a common means for discerning the overwintering activity and locations of migratory species in terrestrial‐based habitats, especially stable isotope ratios of 2‐hydrogen (deuterium, δ^2^H), 13‐carbon (δ^13^C), and 15‐nitrogen (δ^15^N) (Hobson, 1999; Norris, Marra, Kyser, & Ratcliffe, 2005; Yerkes, Hobson, Wassenaar, Macleod, & Coluccy, 2008). Isotopic signatures reflect the environment in which a given tissue (and by extension the individual) grows (Bearhop, Waldron, Votier, & Furness, 2002; Bond & Jones, 2009) because these stable isotopes are integrated within an individual's tissues through consumption of locally acquired water and food. Therefore, by matching tissue‐specific isotopic signatures to terrestrially delineated isotopic landscapes called isoscapes, stable isotopes have been successfully used to determine the overwintering or breeding areas of many terrestrial birds (Haché, Hobson, Villard, & Bayne, 2012; Hobson, 1999). Isoscapes of δ^2^H are generated as a result of predictable, regionally generalized patterns of precipitation (Bowen, Wassenaar, & Hobson, 2005; Mehl, Alisauskas, Hobson, & Merkel, 2005) and have proven useful for inferring the terrestrial overwintering grounds of various migrant bird species (Haché et al., 2012; Hénaux, Powell, Vrtiska, & Hobson, 2012; Hobson, Bowen, Wassenaar, Ferrand, & Lormee, 2004; Yerkes et al., 2008). Unlike deuterium, δ^13^C and δ^15^N isoscapes are generated by landscape‐scale processes related to nitrogen cycling in the soil (δ^15^N) and the plant types present (δ^13^C) (Bond & Jones, 2009; Rubenstein & Hobson, 2004). Recently, δ^13^C and δ^15^N have been combined together to infer the overwintering locations of migratory shorebirds sampled at coastal stopover sites along western Africa and Europe (Catry et al., 2016). Thus, it is possible to use multiple isotopes to assign individuals to a more specific location. For example, δ^2^H, δ^13^C, and δ^15^N were used to determine the staging and overwintering areas of Alaskan northern pintails (*Anas acuta;* Yerkes et al., 2008), as well as the natal origins of five different species of European bat species (Popa‐Lisseanu et al., 2012).

The ability to apply stable isotope analysis to marine species is generally more challenging than terrestrial systems due to the highly dynamic nature of marine systems, which results in less predictable isoscapes, making isotope ratios collected from seabirds more difficult to define and interpret (Bond & Jones, 2009). To overcome the lack of reliable isoscapes in the marine environments, integrative ecologists have begun comparing the isotopic values from tissues in the species of interest with those occupying lower trophic positions (Mehl et al., 2005) as a proxy for the isotopic signal of the environment. For example, δ^15^N was used to infer the overwintering locations of breeding king eiders (*Somateria spectabilis*), by matching δ^15^N values in eider feathers, which were grown in winter, to signatures in copepod prey collected from two known overwintering areas located ~3,000 km apart (Mehl et al., 2005). A limitation to this approach arises when comparing dissimilar tissues types (e.g., blood versus feathers), or when making interspecific comparisons, as tissue‐ or species‐specific discrimination factors are required to accurately relate the isotopic signatures of an animal to that of its prey (Bearhop et al., 2002; Bond & Diamond, 2011). This is because the incorporation of isotopes into different tissues varies as a function of their structural composition and/or turnover rate, and because incorporation rates can also vary among species with differing metabolic rates, energetic requirements, and life histories (Bearhop et al., 2002; Haché et al., 2012). Discrimination factors that account for these factors are required to make meaningful interpretations; however, they are often not available or quantifiable for a given study. A potential solution for determining the nonbreeding, winter location of a species is to characterize the isotopic signatures using specific tissues from individuals collected at the known wintering areas (Norris et al., 2005). This method has the advantage of negating the need for discrimination factors as well as providing a baseline wintering reference signature to which samples collected from other individuals at a different time and location (e.g., on the breeding grounds) can be compared.

**Figure 1:**
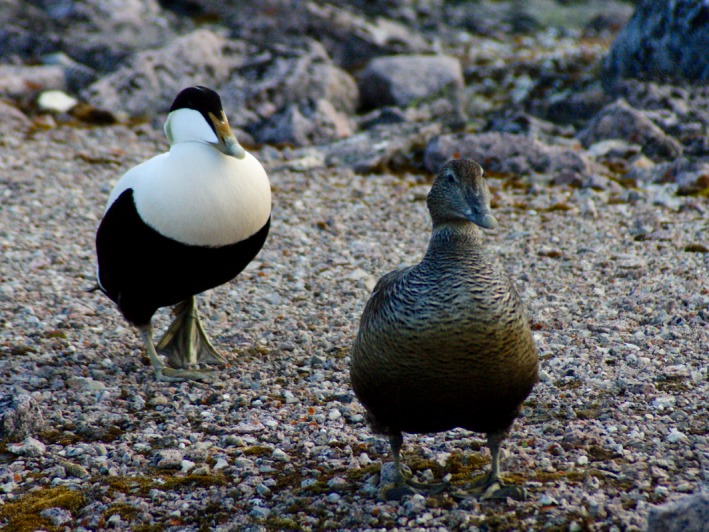
A pair of common eiders on East Bay Island, Nunavut (Photograph: R. Steenweg)

In this study, we identify wintering δ^13^C, δ^15^N, and δ^2^H isotope values for the northern common eider (*Somateria mollissima borealis,* Figure 1.; hereafter “eider”), a migratory sea duck which spends a majority of its life on the ocean, with the aim to assign overwintering locations to individuals sampled at arrival on breeding grounds. Previous satellite tracking studies at the breeding colony of our study site (East Bay Island, Nunavut, Canada) have indicated that eiders migrate to, and spend their winter in, two general locations: off the southwestern coast of Greenland near Nuuk and northwards toward Disko Bay, and along the coast of Newfoundland and Labrador, Canada (Mosbech et al., 2006). Beginning in late April, eiders leave their overwintering areas for the staging areas in and around northern Hudson Bay, arriving there in late May traveling between 60 and 130 km per day (Mosbech et al., 2006). Eiders move to the breeding colony in early to mid‐June when the ice has begun to clear from the head of the bay (F. Jean‐Gagnon, unpublished data). The two wintering locations are markedly distinct with regard to their geology, community composition, and hydrology and thus provide an amenable system in which to test predictions about isotopic differentiation in eider tissues, and make inferences about geographic distribution during the nonbreeding period in winter. Specifically, we first predicted that the δ^15^N signatures of eiders overwintering in Greenland would be greater than in birds from Newfoundland due to known differences in local circulation and nutrient enrichment patterns between the Labrador and West Greenland currents, and greater δ^15^N enrichment in Greenland (Rubenstein & Hobson, 2004). Second, because a large proportion of adult eiders in Greenland spend most of the winter in fjords while eiders in Newfoundland move along coastal areas and offshore islands, and δ^13^C differs with distance from shore and as a function of latitude (Cherel et al., 2008; Rubenstein & Hobson, 2004), we predicted that δ^13^C would be higher in birds overwintering in Greenland (Graham, Koch, Newsome, McMahon, & Aurioles, 2010). Finally, as precipitation is directly related to δ^2^H levels (Bowen et al., 2005; Mehl et al., 2005), we predicted that the substantial freshwater inputs from melting glaciers in Greenland would result in lower δ^2^H levels in Greenland compared to Newfoundland (Bowen, 2010).

Studies using stable isotopes to assign overwintering sites for birds often use isotopes obtained from feather samples (Garcia‐Perez & Hobson, 2014; Hobson, Van Wilgenburg, Wassenaar, & Larson, 2012; Mehl et al., 2005). However, eiders undergo a near‐complete postbreeding molt in the fall, before migrating to their overwintering locations, meaning these signatures would reflect molting rather than wintering sites. Therefore, after investigating differences in isotopic values of common eider populations, our second goal was to test whether blood or claw (toenail) tissues would be best used to infer overwintering origin of arriving eiders.

Our third goal was to determine whether we could assign unknown individuals arriving on the breeding grounds to specific wintering locations using a *k*‐means clustering method. Specifically, we tested several *k*‐means clustering analyses using different combinations of the stable isotope data, to best classify our known winter eider samples to their correct overwintering location. Subsequently, we included samples collected from prebreeding eiders in these clustering analyses to assign overwintering locations to prebreeding eiders based on their isotopic signatures. With these results, we can discuss the resulting proportions of prebreeding eiders assigned to either the Greenland or Newfoundland overwinter groups and compare these to the proportions expected from previous telemetry studies in our system.

## 2 METHODS

### 2.1 Study system

**Figure 2:**
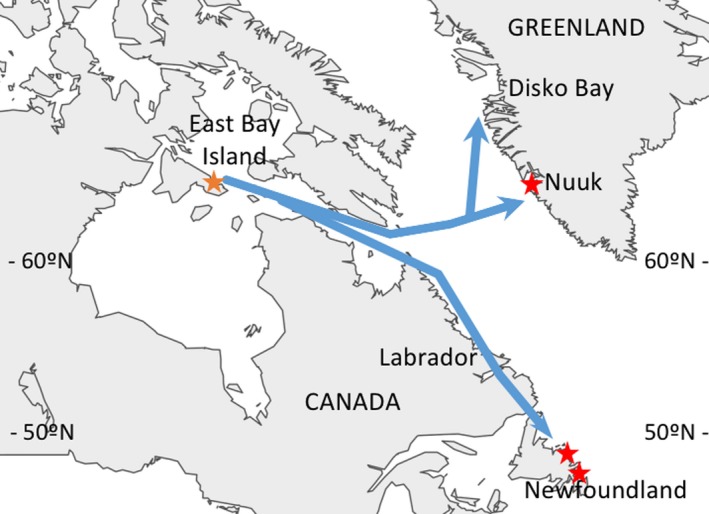
Map of eider migration from the breeding colony at East Bay Island to overwintering areas in Greenland and Newfoundland. Winter sampling sites are denoted with red stars and East Bay Island with an orange star

To characterize the isotopic makeup of each overwintering location, eiders were sampled on their overwintering grounds (Figure 2). Eiders in Newfoundland (Change Islands; 49°57′N, −54°27′W) were collected by hunters and submitted to Environment and Climate Change Canada (Mt. Pearl, NL) between 23 December 2013 and 17 January 2014 for a contaminants study. Additional eiders were collected from Newfoundland (Sunnyside; 47°48′N, −53°53′W), when several died after striking light standards at a coastal industrial site on 01 April 2016 and were submitted to Environment and Climate Change Canada. In Greenland (Qussuk Fjord, Nuuk; 64°76′N, −51°01′W), local fishermen collected eiders from fisheries bycatch between 15 April and 22 April 2014 and submitted them to the Greenland Institute of Natural Resources. Any eiders showing signs of decomposition or oiling were not sampled for this study. All eider carcasses were frozen at −20°C until dissection. Additional details about these collections, including sample sizes and the types of tissues collected, are summarized in Table 1. Arriving, prebreeding eiders used to assign to wintering groups were captured at their breeding colony, East Bay Island (EBI), East Bay Migratory Bird Sanctuary, Nunavut, Canada (Figure 1; 64°02′N, 81°47′W), using flight nets during the prebreeding period (11 June to 01 July 2014 and 19 June to 04 July 2015).

**Table 1:** Summary of samples collected from each location, the time period that tissues will reflect isotopically, and sample sizes (*N*)

Location	Tissue	Date of collection	Time period reflected	*N*	*N* males	*N* females
Newfoundland	Claws	April 2016	Winter	24	8	16
	Whole blood	December 2013 to January 2014	Winter	35	30	5
Nuuk, Greenland	Claws	April 2014	Winter	33	6	29
	Whole blood	April 2014	Late winter	34	6	29
East Bay Island	Claws	June to July 2014	Winter	109	0	109
	Red blood cells	June to July 2014	Spring migration	108	0	108
	Claws	June to July 2015	Winter	115	43	72
	Red blood cells	June to July 2015	Spring migration	125	51	74

### 2.2 Tissue sample collection

The time period reflected by a specific tissue depends on the tissue's turnover or growth rate (Hénaux et al., 2012; Oppel & Powell, 2010; Steenweg, Ronconi, & Leonard, 2011). In birds weighing ~1.5 kg, whole blood and red blood cells have a turnover rate of approximately 3–4 weeks (Hahn, Hoye, Korthals, & Klaassen, 2012). Claws, however, do not have a turnover rate because they are metabolically inert and growth is continuous, and, for a mature duck, a typical claw will represent ~90–110 days of growth (Hopkins, Cutting, & Warren, 2013), as the tip of the claw is filed down by natural abrasion. Therefore, in eiders sampled on their wintering grounds, the base of the claw should reflect the most recent growth and the most accurate wintering signature. For eiders collected at the breeding grounds, the full claw length, from tip to base, would reflect growth over the previous 3 months, which overlaps with their time spent within their core overwintering areas (Mosbech et al., 2006).

For claw samples collected from overwintering birds, total claw length was measured for each individual, and samples were clipped from the base of the claw on the middle toe of the left foot and stored in a paper envelope. For birds captured at the breeding site, the middle toe claw of the left foot was measured from base to tip to the nearest millimeter, and the distal 2 mm of the claw was clipped and stored in a small paper envelope for further analysis.

We also collected blood samples from wintering eiders to compare isotope levels between locations and within locations across years. Blood samples for wintering birds were collected from Newfoundland (Change Islands) and Nuuk, Greenland. Frozen whole blood was removed from the heart atrium or ventricle and stored in an Eppendorf tube for further analysis. For prebreeding eiders at EBI, fresh 1 ml blood samples were taken from the tarsal vein using a heparinized 23‐gauge needle and syringe, then stored in heparinized Eppendorf tubes and kept cool to approximately 4°C. This blood was collected as part of another project, and therefore, all samples were centrifuged at 6,700 g for 10 min, and red blood samples were separated from plasma, unlike in our wintering birds. Red blood cells were stored at −80°C until prepared for analysis.

### 2.3 Laboratory analyses

To prepare samples for stable isotope analysis, blood samples were oven‐dried at 50°C for 30 hr (winter eiders) or freeze‐dried for 30 hr (breeding site samples). Although samples were dried using different methods, these two drying techniques have been shown to have no effect on stable isotope analysis results (Hobson, Gloutney, & Gibbs, 1997). All blood samples were lipid‐extracted to reduce the effect additional lipids may have on the δ^13^C signatures (Mazerolle & Hobson, 2005), making whole blood and red blood cell samples comparable, and we removed surface oils from claw samples. To extract lipids and remove surface oils, all dried blood and claw samples were soaked in 2:1 chloroform:methanol solution (C:M) for 24 hr and then centrifuged for 10 min at 10,000 rpm. The C:M was siphoned off using a pipette, and then samples were rinsed again with C:M and centrifuged for an additional 10 min, and the C:M was siphoned off once more. Samples were then left open under a fume hood for 24 hr to allow any leftover C:M to evaporate. All blood samples were ground with a mortar and pestle into a powder, and claw samples were snipped into tiny pieces. Subsamples were weighed to 0.3–0.5 mg and folded into a tin capsule for δ^13^C and δ^15^N analysis, and for δ^2^H analysis, subsamples were calibrated in the laboratory for 48 hr, weighed to 0.1–0.2 mg, desiccated in an oven at 100°C for 1 hr, and crushed into a silver capsule. Results of stable isotope analyses are reported in δ units where δ = [(R_sample_/R_standard_) − 1] × 1,000. R_sample_ are the ratios of the isotopes (i.e. C^13^/C^12^, N^15^/N^14^, and H^2^/H^1^) in samples, and R_standard_ are the ratios of isotopes in the international standards, unique for each element (for carbon: Vienna Pee Dee Belemnite, nitrogen: Atmospheric Air, hydrogen: Vienna Standard Mean Ocean Water). Standards were run every five samples and duplicates were analyzed for every nine samples, with precisions of 0.2‰ for δ^13^C and δ^15^N analysis and 3‰ for δ^2^H (Macdonald et al., 2012; Norris et al., 2005). Stable isotope analyses were performed at the Queen's Facility for Isotope Research, Queen's University (Kingston, ON, Canada) using a Costech ECS 4010 for δ^13^C and δ^15^N analysis and a Thermo Finnigan thermo‐combustion elemental analyzer for δ^2^H analysis coupled to a Thermo Finnigan DELTA^plus^ XP Continuous‐Flow Isotope Ratio Mass Spectrometer.

### 2.4 Data analyses

To address our overarching goal and test our initial hypotheses that δ^13^C, δ^15^N, and δ^2^H values in blood and claws will differ between Greenland and Newfoundland samples, we ran separate two‐factor ANOVA models to compare δ^13^C, δ^15^N, and δ^2^H values from wintering birds, using location and sex as factors. We ran a one‐factor ANOVA for each isotope in eiders collected at East Bay in 2014 and 2015 to test for differences between years. There was no discernable annual variation in claw isotopes, and thus we pooled these tissues from 2014 to 2016 for the wintering birds.

To address our second goal—to determine whether blood or claws are the best tissue to use for these analyses—we assessed whether stable isotopes of these samples overlapped for overwintering and arriving eiders. The turnover rate for blood in birds of this size (Bearhop et al., 2002; Hahn et al., 2012; Steenweg et al., 2011) may be too rapid to be used in this circumstance. If this is the case, we would perform our *k*‐means cluster analyses using the stable isotope signatures from claws.

To address our third goal of determining which isotopes are best included in the *k*‐means clustering algorithm for later assigning an arriving eider to its overwintering site, we tested a *k*‐means clustering method for its ability to correctly classify known winter‐sampled eiders to their correct overwintering location based on the stable isotope signatures in claws. This *k*‐means approach is a centroid‐based partitional clustering method, where the centroids are the arithmetically calculated centers of the clusters and *k* is the number of clusters. The initial centroids for each cluster can either be randomly selected or pre‐assigned from the data (Tan, Steinbach, & Kumar, 2006). Each of the remaining data points is iteratively assigned to the cluster to minimize the sum of squared error of each centroid (Tan et al., 2006). This method has been used previously on stable isotopes and other biomarker data to classify passerines (Garcia‐Perez & Hobson, 2014) and marine mammals into discrete groups (Pomerleau, Lesage, Winkler, Rosenberg, & Ferguson, 2014). We defined the starting centroids from the means of isotope values obtained from individuals in each overwintering location because previous studies indicate that eiders breeding at East Bay Island overwinter in two distinct regions (Mosbech et al., 2006; Figure 2). The western Greenland overwintering group primarily overwinters near Nuuk; however, some migrate 600 km further north to Disko Bay. Because exploratory plots indicated a third potential group, we ran cluster analyses with both two and three clusters for both years, using the mean of the third group as the starting centroid for this new cluster. We conducted cluster analysis for each year separately because the presence of this third group varies between years.

Having predefined the starting centroids for the cluster analysis, we then ran the known identity, winter‐sampled individuals through the cluster analysis to determine whether the analysis could correctly classify individuals to their original location/group. We elected to test *k*‐means cluster analysis using all the stable isotopes (1) δ^13^C, δ^15^N, and δ^2^H, (2) δ^13^C and δ^15^N together, and then (3) only δ^13^C as two‐way ANOVA results indicated that there was only a significant difference between groups in δ^13^C but not δ^2^H or δ^15^N (Table 2). We used the sum of the squared error to measure the quality of each clustering method (Tan et al., 2006), used the number of misclassified winter birds as a measure of its accuracy, and plotted the results to determine whether the clustering was realistic. All data analyses were conducted using R version 3.3.1 (2016‐09‐28) using packages cluster (Maechler, Rousseeuw, Struyf, & Hubert, 2016) and MASS (Venables & Ripley, 2002).

**Table 2:** Two‐way ANOVA model results for isotope signatures in eiders sampled from the two overwintering areas for both whole blood and claws

Tissue	Isotope	Means ‰ (*SD*)		*df*	*F* value	Overall	Location	Sex
		Nuuk, Greenland	Newfoundland			*p*	*p*	*p*
Whole blood	δ^13^C	−18.55 (0.90)	−20.05 (0.51)	2, 67	35.89	**<.001**	**<.001**	.792
	δ^15^N	10.19 (0.43)	10.88 (0.60)	2, 67	15.64	**<.001**	**<.001**	.709
	δ^2^H	−78.85 (6.91)	−71.26 (7.33)	2, 67	11.6	**<.001**	**<.001**	.171
Claws	δ^13^C	−18.12 (0.60)	−20.55 (0.58)	2, 55	119.6	**<.001**	**<.001**	.632
	δ^15^N	12.98 (0.77)	13.14 (0.52)	2, 55	0.352	.704	.414	.987
	δ^2^H	−49.97 (9.27)	−50.33 (7.04)	2, 55	0.221	.803	.785	.522

Significant relationships are bolded.

## 3 RESULTS

The δ^13^C, δ^15^N, and δ^2^H signatures from blood samples of the two groups of winter‐caught eiders (Greenland and Newfoundland) were significantly different (*p *<* *.001 for each), but did not differ by sex (Table 2). Likewise, claw δ^13^C signatures differed significantly between the two groups (Table 2; *p *<* *.001), although δ^15^N and δ^2^H signatures were not significantly different (Table 2; *p *=* *.70 and *p *=* *.80, respectively) and sex was not a significant factor (Table 2). Moreover, although we did not detect any annual variation in δ^13^C signatures of claws from birds captured at arrival in 2014 and 2015 (*F*_1, 222_ = 1.90, *p *=* *.17), both δ^15^N and δ^2^H signatures differed significantly across years (δ^15^N: *F*_1, 222_ = 6.29, *p *=* *.013; δ^2^H: *F*_1, 217_ = 6.36, *p *=* *.012).

**Figure 3:**
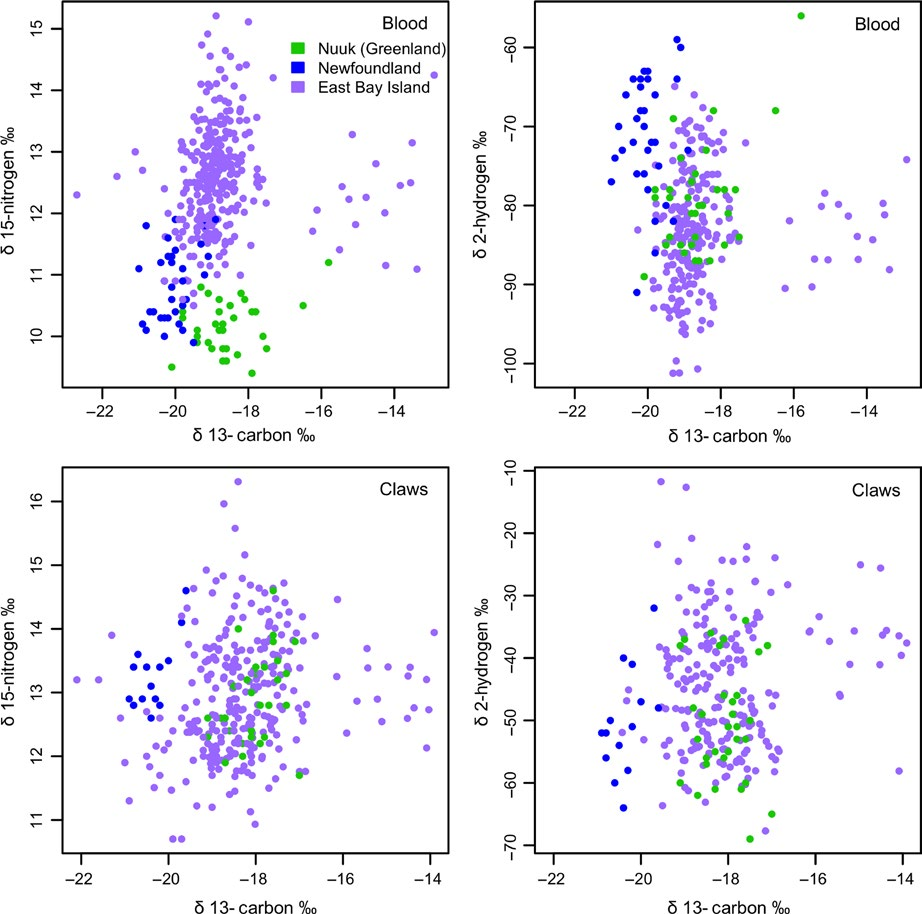
Scatterplot of stable isotope data for winter (Nuuk, Greenland, and Newfoundland)‐ and prebreeding (East Bay Island)‐caught eiders for both blood and claw tissues

Stable isotope signatures in the blood samples of eiders arriving at the breeding colony overlapped with those from wintering eiders from Newfoundland, but not with the wintering eiders from Greenland (Table 3; Figure 3). Moreover, signatures from prebreeding birds were comparatively enriched in δ^15^N and depleted in δ^2^H (Table 3; Figure 3), suggesting they partially reflected signatures acquired during the migration period. Consequently, we used the stable isotope signatures from claws of prebreeding eiders to conduct *k*‐means cluster analysis.

**Table 3:** Summary of stable isotope signatures in eider blood and claws from individuals sampled during the prebreeding period at East Bay Island

Tissue	Isotope	2014			2015		
		Means δ‰ (*SD*)	Min δ‰, Max δ‰	*N*	Means δ‰ (*SD*)	Min δ‰, Max δ‰	*N*
Blood	δ^13^C	−18.17 (1.59)	−19.77, −12.91	108	−18.87 (0.52)	−20.28, −17.32	125
	δ^15^N	12.38 (0.66)	10.60, 14.25	108	12.91 (0.92)	10.92, 15.21	125
	δ^2^H	−80.02 (6.61)	−94.99, −64.93	107	−86.10 (6.57)	−101.23, −70.06	121
Claws	δ^13^C	−17.92 (1.46)	−20.43, −13.91	109	−18.13 (0.78)	−20.28, −15.68	115
	δ^15^N	12.90 (0.73)	11.47, 14.69	109	13.20 (1.07)	10.93, 16.31	115
	δ^2^H	−42.30 (10.73)	−63.11, −11.75	106	−45.76 (9.72)	−67.70, −20.83	112

**Figure 4:**
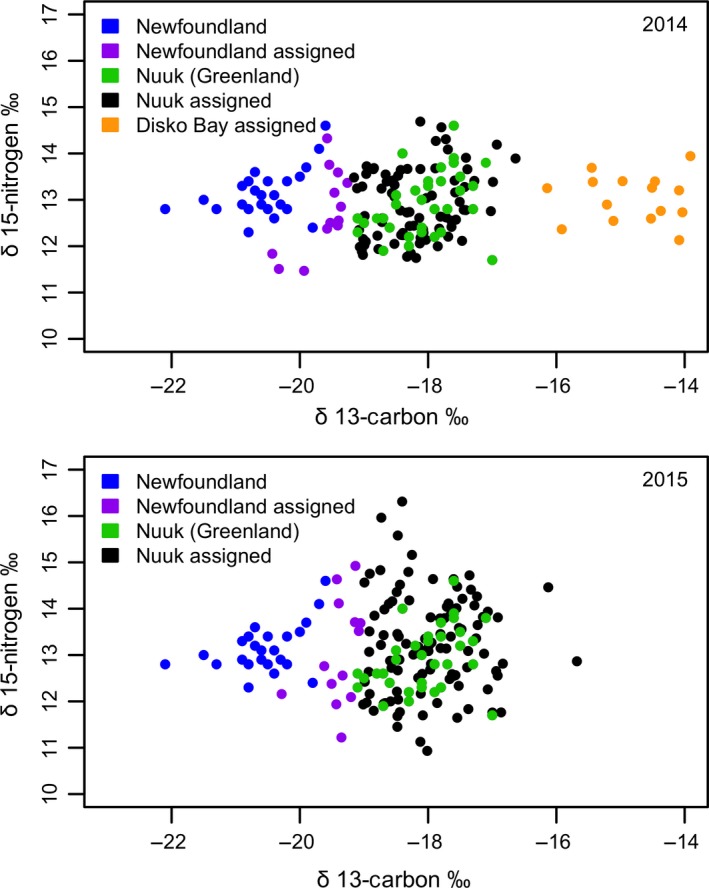
Scatterplot of the results of one‐dimensional *k*‐means cluster analysis using the stable isotopes of carbon found in claws obtained from eiders during the prebreeding periods in 2014 and 2015 and from their overwintering sites in Newfoundland, Canada, and Nuuk, Greenland. “Assigned” refers to prebreeding eiders assigned to their respective overwintering areas. Results are plotted against nitrogen for ease of visualization

Tests of *k*‐means cluster analyses using the stable isotopes found in the claws of winter‐caught eiders minimized *SSE* and resulted in fewer misclassified wintering eiders when using the stable isotope δ^13^C alone (2014: 0 and 2015: 2 misclassified) as opposed to integrating δ^13^C and δ^15^N (2014: 0 and 2015: 3 misclassified), or δ^13^C, δ^15^N, and δ^2^H values (2014: 29 and 2015: 28 misclassified; Table 4). For 2014 prebreeding eiders, the most parsimonious representation of groupings included three clusters (8 vs. 0 misclassified for 2 vs. 3 clusters, respectively), where for the 2015 prebreeding eiders, two clusters yielded the best results (2 vs. 16 misclassified for 2 vs. 3 clusters, respectively; Table 4). These two models suggest that in 2014, a total of 79 individuals overwintered in Nuuk, Greenland, 13 in Newfoundland, and another 15 near Disko Bay, Greenland, while in 2015 it was estimated that 102 eiders overwintered in Nuuk, Greenland, and 13 in Newfoundland (Figure 4).

**Table 4:** Assessment of strength of each *k*‐means cluster analysis using total variance explained and number of misclassified winter birds

Year	*K*	Stable isotopes included	Between sum of squares	Total sum of squares	Total Variance Explained (%)	Final centroids (δ^13^C ‰, δ^15^N ‰, δ^2^H ‰)			Number of misclassified winter birds
						1	2	3	
2014	3	δ^13^C, δ^15^N, δ^2^H	14770.32	18794.45	78.59	−18.83, 12.74, −56.70	−18.50, 12.87, −45.54	−17.56, 13.24, −32.07	29
	3	δ^13^C, δ^15^N	322.26	459.60	70.16	−18.15, 12.90	−20.20, 13.02	−14.81, 13.04	0
	**3**	**δ** ^**13**^ **C**	321.79	382.35	**84.16**	−18.16	−20.23	−14.81	**0**
	2	δ^13^C	196.29	382.35	51.34	−17.24	−19.45	NA	8
2015	2	δ^13^C, δ^15^N, δ^2^H	10726.89	15361.29	69.83	−18.51, 12.88, −53.24	−18.41, 13.61, −36.42	NA	28
	2	δ^13^C, δ^15^N	135.92	362.01	37.54	−17.93, 13.23	−19.93, 12.90	NA	3
	**2**	**δ** ^**13**^ **C**	133.88	208.94	**64.08**	−17.97	−20.09	NA	**2**
	3	δ^13^C	176.74	208.94	84.59	−18.65	−20.50	−17.51	16

Those resulting in the fewest misclassified winter birds are bolded. Final centroid refers to the mean (or center) of the clusters formed by the *k*‐means cluster analysis, which differ from the starting centroids used to guide the beginning of the analysis.

## 4 DISCUSSION

Supporting our first goal, isotopic signatures were indeed significantly different between the two wintering groups. In contrast to a study on American redstarts (*Setophaga ruticilla*; Norris et al., 2005), blood samples did not reflect overwintering sites, but rather signatures probably obtained during spring migration likely due to the slow nature of eider migration to the breeding grounds at East Bay Island. Cluster analysis of stable isotope values in the claws of eiders, however, was more successful in differentiating between wintering locations, and using this technique, we were then able to infer the overwintering locations of eiders arriving at their breeding colony, which supports our second goal. Concerning our third goal, δ^13^C alone, and not the integration of δ^13^C with δ^15^N and δ^2^H, was most useful for differentiating wintering locations, likely because these areas differ in habitat features known to affect δ^13^C values (marine coastal vs. inland fjord), and in latitude (Cherel et al., 2008; Graham et al., 2010; Rubenstein & Hobson, 2004).

*K*‐means cluster analysis was effective for determining both the number of clusters to include for each year (2014 or 2015) and their respective centroids. Moreover, the method revealed a novel overwintering group and generated very few misclassified winter birds. Discriminant function analysis (DFA) is sometimes similarly used in this circumstance, but is not recommended with the use of spatial data where as clustering methods are (Zuur, Leno, & Smith, 2007). In comparison with DFA, *k*‐means cluster analysis uses starting centroids to form the clusters, rather than DFA which provides limits from which the groups or clusters are formed. Secondly, because winter samples from the third inferred group from Disko Bay were unavailable, we did not have the boundaries to include in the DFA. Including the mean of this group as a starting centroid for *k*‐means was a way to manage this issue. Nonetheless, one advantage of DFA over *k*‐means cluster analysis is that DFA can assign a percentage of confidence to each individual's assignment; however, given the infrequent misclassification of winter birds, we are confident that *k*‐means cluster analysis of δ^13^C adequately classified arriving eiders into their known overwintering groups. Overall, this work demonstrates that it is possible to not only back‐assign individual eiders to their overwintering grounds using samples collected upon arrival at their breeding grounds, but also to detect potentially novel wintering grounds.

Although blood samples were obtained in January and April from Newfoundland and Greenland, respectively, we expected differences in isotopes to be applicable as samples were obtained within 4 months of each other, and the differences between locations would be bigger than between the different times. The differences between the two overwintering areas in blood and claws were similar, although a bit larger for claws. As such, we are confident that the differences in blood were due to their geographic location rather than the differences in timing.

The ability to back‐assign seabirds and sea ducks arriving at the breeding colony to their overwintering locations is a major advance for studies of marine birds. This method has the potential to significantly impact future studies investigating the carryover effects of migration behaviors on individual‐ and population‐level processes in a number of ways. First, future studies using these techniques can cost‐effectively and relatively noninvasively determine overwintering location and therefore determine how variation in overwintering environmental conditions carryover to affect important reproductive parameters. Second, once established as a baseline, this method can then be used to delineate populations and monitor any fluctuations in these populations. For example, previous satellite tracking research (2001–2003) from this colony indicated that approximately 40% of eiders overwintered in Newfoundland, Canada, and 60% in Greenland (Mosbech et al., 2006). We found that in both years, only about 10% of the eiders overwintered in Newfoundland, with different proportions arriving from Nuuk and Disko Bay, Greenland, in each year. In 2014, 14% of the eiders were detected to occupy a third overwintering cluster (Table 4). We suggest that these individuals are likely from Disko Bay, because more northern marine areas are enriched in 13‐carbon (Hobson, 1999; Rubenstein & Hobson, 2004; West, Bowen, Cerling, & Ehleringer, 2006); therefore, δ^13^C is higher in Disko Bay compared to Nuuk as seen in shrimp (*Pandalus borealis*) and copepods (*Calanus finmarchicus*; Hansen, Hedeholm, Sünksen, Christensen, & Grønkjær, 2012). While the western Greenland breeding population has increased by 12% per year (Merkel, 2010) since the implementation of hunting quotas, the Newfoundland population has been potentially impacted by the same Avian Cholera outbreak that affected East Bay Island and other colonies along the Hudson Strait (Iverson, Forbes, Simard, Soos, & Gilchrist, 2016). This shift in proportion of eiders overwintering in Greenland versus Newfoundland may therefore be influenced by spillover from the Greenland population. Alternatively, the changes in proportions may be more reflective of the relatively small sample size (*n *=* *25) from the satellite tagging study compared to this study. This emphasizes the importance of using isotopic methods to track changes in population demographics and migration patterns. This could be especially important as climate change pushes animals out of traditional ranges or to become nonmigratory; new isotopic signatures in breeding individuals could indicate novel overwintering sites and direct future winter sampling.

### 4.1 Implications for future applications

Using stable isotope analysis as an indirect tracking method, rather than direct tracking methods using instruments (e.g., satellite or GPS telemetry, geolocation.), has the potential to provide meaningful location data while also allowing for larger sample sizes at substantially lower costs and simultaneously reducing the impact on the individual animals being tracked (Bowlin et al., 2010). Although the use of stable isotopes to infer overwintering origin is quite common in terrestrial species (Garcia‐Perez & Hobson, 2014; Haché et al., 2012; Hobson et al., 2004, 2012; Miller, Wassenaar, Hobson, & Norris, 2012; Popa‐Lisseanu et al., 2012; Rubenstein & Hobson, 2004; Yerkes et al., 2008), individuals are often assigned by comparing isotope signatures in feathers to established carbon‐ or hydrogen‐based isoscapes. It is difficult to tailor this method for use in marine species, however, the more infrequently used approach of sampling individuals from their overwintering and breeding sites, and then subsequently testing different tissues and isotope combinations can be applied across taxa. Alternatively, we suggest that this method could also be easily adapted to infer breeding areas to flocks of overwintering birds, if samples are collected at appropriate times.

Oppel and Powell (2008) provide an example of using head feathers from king eiders in the western Arctic to assign individuals to overwintering areas. In marine birds where specific feather molt schedules may be unknown and in those species that undergo a near‐complete full‐body molt in the fall (Goudie, Robertson, & Reed, 2000), claws are well‐suited tissues to sample to reflect overwintering signatures in both winter‐ and prebreeding‐caught individuals. Claws have a growth rate that is useful for both time periods, sampling is entirely noninvasive and will not impact flight like sampling flight feathers may (Swaddle, Witter, Cuthill, Budden, & McCowen, 1996), and they are easily collectable and therefore simple to include in sampling protocols. In addition, this method allows one to avoid having to use discrimination factors, which are required when comparing different tissues, and therefore reduces the ambiguity in our location estimations (Bearhop et al., 2002; Bond & Diamond, 2011). The use of 13‐carbon in claws also simplifies the technique, as studies looking at 2‐hydrogen need to consider the exchange of water within tissues (Hobson, Atwell, & Wassenaar, 1999).

For studies on marine birds especially, which are often long‐lived and faithful to both their overwintering and breeding sites (Mallory, Gaston, Gilchrist, Robertson, & Braune, 2010; Reed, Harris, & Wanless, 2015; Robertson & Cooke, 1999), we suggest that researchers combine their tracking studies with analysis of multiple stable isotopes so that they can ground‐truth stable isotope tracking methods simultaneously with telemetry tracking. In addition, researchers could use isotopes as a way of increasing their sample size to test whether the individuals they have specifically tagged are representative of the overall population. This would be especially useful in population delineation studies, a focus in marine bird research and among wildlife managers (Boyd, Bowman, Savard, & Dickson, 2015; Gilliland et al., 2009). The only drawback of the aforementioned tracking study of this breeding colony (Mosbech et al., 2006) is that the eiders were not sampled for stable isotopes in conjunction with satellite tracking, and therefore, we could not completely validate our winter assignment for arriving eiders. Ideally, tracked birds would be sampled for stable isotope analysis to test for differences between isotopic signatures in relation to where the tracking devices indicate they spend the period of time of interest to validate isotopic approaches. Of course, this method requires that individuals are faithful to overwintering areas.

We recognize that isotopic baselines can have some isotope‐specific temporal variation (Bowen, 2010; Rubenstein & Hobson, 2004). For instance, atmospheric δ^13^C can fluctuate by as much as 0.75 ‰ in higher latitudes within one year and has shown a total decrease of 0.25–0.5 ‰ over a ten‐year period depending on the latitude (Bowen, 2010) as a result of ever‐increasing CO^2^ emissions (West et al., 2006). In turn, these fluctuations in atmospheric δ^13^C can affect patterns reflected in the oceans. Consequently, we suggest future studies aiming to assign individuals to an overwintering location, resample reference winter locations at least every five to ten years to account for these variations.

In summary, following our study design, we recommend that researchers test the effectiveness of several stable isotopes to determine the best combination for their system. Nevertheless, using these types of isotopic assignment methods could replace costly (in terms of funds and impacts on individual birds) device‐based tracking studies as a viable solution for increasing sample size, delineating populations, and monitoring more populations or species simultaneously. Indeed, these methods should be readily transferable to other life‐history periods (i.e., breeding locations of groups of wintering birds). Further exploratory studies are needed to investigate the feasibility with pelagic seabirds; however, these methods are applicable to other sea ducks and more broadly to coastally feeding seabirds and shorebirds, and other marine animals.

## CONFLICT OF INTEREST

None declared.

## AUTHOR CONTRIBUTIONS

RJS collected and processed samples from all field sites, conducted isotope analyses and data analyses, and wrote the manuscript. GTC helped extensively with data analyses and manuscript writing. TKK provided facilities and training for isotope analyses and provided feedback for interpretation of the data. FRM collected samples from GRLD and helped with development of ideas. HGG collected samples from EBI and helped with conception of ideas. HLH collected samples from EBI, helped with interpretation of data, and helped with sample processing. GJR collected and provided samples from NFLD. JFP collected and provided samples from NFLD. JMF provided extensive feedback and guidance for data analyses and interpretation. OPL collected samples from EBI, came up with initial idea for work, and helped extensively with manuscript writing. All authors critically revised and approved the final version of the manuscript submitted.

## DATA ACCESSIBILITY

Data will be archived in the publicly accessible DRYAD repository.
